# The development and evaluation of a sub-health self-rating scale for university students in China

**DOI:** 10.1186/s12889-019-6650-3

**Published:** 2019-03-21

**Authors:** Jian-lu Bi, Jing Chen, Xiao-min Sun, Xiao-li Nie, Yan-yan Liu, Ren Luo, Xiao-shan Zhao

**Affiliations:** 1 Endocrinology Department, Guangdong Second Traditional Chinese medicine Hospital, Guangzhou, 510095 Guangdong China; 2 School of Traditional Chinese Medicine, Southern Medical University, Guangzhou, 510515 Guangdong China; 3 Hospital of Guangdong University of Technology, Guangdong University of Technology, Guangzhou, 510006 Guangdong China

**Keywords:** Sub-health, Questionnaire, Reliability, Validity, University students

## Abstract

**Background:**

Sub-health status is defined as declines in vitality, physiological function and capacity for adaptation, but without the presence of clinical or sub-clinical disease. We have developed and evaluated a comprehensive questionnaire, the Sub-Health Self-Rating Scale (SSS), to assess sub-health status in university students.

**Method:**

The items for the draft questionnaire were discussed in focus groups. The WHOQOL-BREF was selected as the validity reference. From a professional perspective and large sample evaluation, the scale ultimately consisted of 58 items. The reliability and validity of the SSS was examined in undergraduate students and 1000 questionnaires were randomly selected from the samples for expert evaluation.

**Results:**

Cronbach’s α of the total scale was 0.942. The dimensions of physiological, psychological and social had high reliability: 0.915, 0.856 and 0.850, respectively. Based on scree plot and related theories, there were 10 factors to be extracted. The correlation coefficient between the total scale and sub-scale was high. The dimensions of physiological, psychological and social had high correlations with the total scale: 0.929, 0.803 and 0.774, respectively. The sub-health cut-off point of the total scale was 72; for the physiological field, it was 72; for the psychological field, it was 60; and the social field, it was 56. The fit between the expert evaluation method and the scale method was 0.758. The lower the score, the worse the health condition.

**Conclusion:**

We established and evaluated a valid instrument (SSS) that encompasses physiological, psychological and social factors to investigate sub-health status. It is short and easy to complete, and therefore suitable for use with undergraduate students.

**Electronic supplementary material:**

The online version of this article (10.1186/s12889-019-6650-3) contains supplementary material, which is available to authorized users.

## Background

The World Health Organization (WHO) defined health in its broader sense in 1946 as “a state of complete physical, mental, and social well-being and not merely the absence of disease or infirmity” [1] . Although this definition lacks operational value, it remains the most enduring of all definitions. With greater understanding of health, the definition has deepened to take account of sub-health status (SHS), which is an intermediate state between disease and health, as proposed by Wang [2].

In China, as a result of rapid economic development, many people face pressures from work and home lives, perhaps working overtime or through the night. As a result, the body clock is affected, the immune system declines [3, 4], and some appear with SHS [5] . TCM clinical guidelines for sub-health released by the China Association of Chinese Medicine pointed out that the SHS is one that shows declines of vitality, physiological function and capacity for adaptation, but it is not defined as clinical or sub-clinical disease [6]. Over the years, the concept of sub-health has been widely accepted in many other countries such as Japan [7], Canada and Australia [8, 9]. For example, a Japanese scholar has conducted a physical check-up of sub-health and investigated the diagnostic criteria for sub-health for Japanese people, and established a comprehensive sub-health examination system, the Complete Sub-Health Check-up, which includes ten major examination items [7].

According to our survey in Guangdong, a sub-health state affected 65.1% of the total survey population [10]. Although the prevalence rate of sub-health is high, there has been a lack of objective clinical diagnostics for sub-health. In addition, its clinical manifestation is complex, subjective and difficult to quantify. It is necessary to develop an objective, simple, self -testing and convenient scale to confirm the status of sub-health.

During such an important period of their lives, undergraduate students repeatedly face changes and challenges. During this developmental stage, a number of students appear with physical, psychological and social problems. Our previous investigations revealed that a number of undergraduates displayed sub-health state [11–13]. It is highly appropriate for undergraduate students to use a self-assessment scale to evaluate their health status.

There are some sub-health state questionnaires already established and evaluated in China, such as SHSQ-25, MSQA and SHMS V1.0 [14–16], but these are not suitable for use with undergraduate students. SHSQ-25 cannot completely measure the physiological, psychological and social aspects and MSQA is aimed at adolescents. As a result, it was essential to develop a reliable and valid instrument to assess SHS. Therefore, we developed and evaluated a comprehensive questionnaire, the Sub-Health Self-Rating Scale (SSS), to assess SHS for undergraduates in China (Additional file 1). It is short and easy to complete, and therefore suitable to use in studies of undergraduate students.

## Methods

### Questionnaire development

The items for the draft questionnaire were identified according to the WHO’s definition of health and Chinese social culture. In addition, we conducted a literature review and solicited expert opinion (comprising two health managers, four physicians, a psychologist and two clinical epidemiologists). The final questionnaire included 83 items.

The second stage was to evaluate the scale. A total of 6000 questionnaires were handed out in a medical university; 5792 questionnaires were handed back (96.53% response rate). Stratified sampling is used to sample at each grade. There is no difference in the age and gender of the sample. The 193 students who did not complete the questionnaires were excluded. Ultimately, 5599 questionnaires were analyzed. Cronbach’s α of the draft questionnaire was 0.949. Cronbach’s α values of the physiological, psychological and social subs-cale were 0.906, 0.881 and 0.865, respectively. Construct validity used exploratory factor analysis (Varimax). According to the value of eigenvalues above 1, we extracted 16 factors (include positive feeling factor, passive feeling factor, pain factor, menstruation factor, sleep factor, capability& self-respect factor, fatigue factor, social relationship factor, urine factor, eyes factor, constipation factor, diarrhea factor, anxious factor, irritable factor, forced factor, hair factor). The cumulative proportion was 52.29%. The Kaiser-Meyer-Olkin (KMO) statistic was 0.960. The criterion validity showed that the scale was highly related to the WHOQOL-BREF (r > 0.60), which was statistically significant (*P* < 0.001). The correlation coefficients between WHOQOL-BREF and the scale were 0.812. There was a notable difference in mean scores between the health and sub-health state in the dimensions (t > 35.00, *P* < 0.000). A total of 63 items were eventually extracted from the draft SSS.

The third stage was to determine the final items. After the draft questionnaire was evaluated, focus groups were again used to discuss whether the items were relevant, clear, unambiguous and written in a language that would be understandable to potential respondents. According to the results of factor analysis, we increased 1 factor (skin factor), put together 2 factors (constipation factor, diarrhea factor) and removed 6 factors (menstruation factor, eyes factor, anxious factor, irritable factor, forced factor, hair factor). From a professional perspective, we deleted nine items, maintained 54 items, and added four items for skin factors. The scale ultimately consisted of 58 items, which had satisfactory sensibility, representation and internal consistency.

### Determining the validity of the questionnaire

#### Study participants

Seven thousands questionnaires were handed out during the 9 month study period; 6232 completed responses were received (89.03% response rate) and 6205 questionnaires were analyzed.

#### Instruments

The SSS was filled in within 20 to 30 min of the student being in the classroom. The students volunteered for our study. Students under 18 years old provided verbal consent from next of kin, carers or guardians. Verbal consent was deemed appropriate because the students volunteered for the study. If they did not want to take part in the questionnaire survey, they could refuse. Secondly, our purpose was to study the health status of the undergraduate students rather than to intervene. All student data were kept in strictest confidence. Although some of the students were under 18, they were mature and understood the aim of the study. The study was approved by the Ethics Committee of Nanfang Hospital in Guangzhou, China [2012] LunShenZi (No. 035). The ethics committee also approved the consent procedure.

There are socio-demographic indicators in the scale, including age, gender, grade, birthplace, marriage status and medical history.

The SSS consisted of 58 items, which were divided into three symptom dimensions (physiological, psychological, social) and ten factors (Table 1). The ten factors were labeled as follows – F1: positive feeling factor, F2:passive feeling factor, F3:pain factor, F4: digestive factor, F5: sleep factor, F6: capability& self-respect factor, F7: fatigue factor, F8: social relationship factor, F9: skin factor, F10: urine factor. Each item had five answer categories corresponding to the degree of each symptom (never, occasionally, sometimes, constantly and always). In the data analysis, never was assigned to 5, occasionally to 4, sometimes to 3, constantly to 2 and always to 1. Then, we added up the scores of all symptoms. The scores of 16 of the symptoms had to be inversely transformed before adding up.

**Table 1:** Theoretical framework for the Sub-health Self-rating Scale (SSS)

Dimension	Factor	Item	Item distribution
Physiological	F5-sleep factor	6	3,8,9,10,11,12
	F7-fatigue factor	6	14,15,16,17,25,26
	F9-skin factor	4	18,19,20,21
	F3-pain factor	7	22,23,24,27,28,29,30
	F4-digestive factor	7	31,32,33,34,35,36,37
	F10-urine factor	3	38,39,40
Psychological	F2-passive feeling factor	6	41,42,43,44,45,52
	F1-positive feeling factor	8	46,47,48,49,50,51,56,57
Social	F6-capability& self-respect factor	4	53,54,55,58
	F8-social relationship factor	4	4,5,6,7
Health evaluation		3	1,2,13
total		58	

#### Expert evaluation

A total of 1000 questionnaires were randomly selected from the valid 6205 cases for expert evaluation to determine the fit between scale and expert evaluation.

### Computational method for the total and sub-scale

The raw score is the sum of the item score in the total scale or the sub-scale. The scores of 16 of the symptoms are inversely transformed before adding up. The conversion formula is as follows:

$$ \mathrm{Converted}\ \mathrm{score}=\frac{\mathrm{raw}\ \mathrm{score}-\mathrm{the}\ \mathrm{lowest}\ \mathrm{possible}\ \mathrm{score}\ \mathrm{in}\ \mathrm{the}\ \mathrm{sub}-\mathrm{scale}\ \mathrm{or}\ \mathrm{total}\ \mathrm{scale}}{\mathrm{the}\ \mathrm{highest}\ \mathrm{possible}\ \mathrm{score}\ \mathrm{in}\ \mathrm{the}\ \mathrm{sub}-\mathrm{scale}\ \mathrm{or}\ \mathrm{total}\ \mathrm{scale}-\mathrm{the}\ \mathrm{lowest}} $$

The linear T score formula is as follows: T = 50 + 10 (*X* + ^−^*X*)/*S*, where *X* is the raw score, ^*−*^*X* is the total mean score, S is the population standard deviation.

#### Statistical analyses

All the data were analyzed using SPSS version 13.0. The reliability verification applied a generalizability coefficient, and the validity verification included factor analysis to verify the validity, criteria validity and distinguish validity. Five methods based on the dispersion, correlation coefficient, factor analysis, Student’s *t*-test and Cronbach’s α were employed to analyze the items. The scale was conducted feasibility analysis. Exploratory and confirmatory factor analysis of 1-order factor and 2-order factor were used to evaluate the infra-structure of the scale. The reliability analysis included Cronbach’s α, split-half reliability and the mean inter-item correlation. The validity analysis included content and construct validity.

## Results

### Characteristics of participants

A total of 6205 university students from grades 1 to 5 were recruited to participate in the survey. The exclusion criteria for participants included having organic disease diagnosed in a clinical laboratory examination. The 176 students, whose medical reports were abnormal, were excluded. Ultimately, 6029 students (2606 male and 3423 female), aged 15 to 29 years (mean age 20.88 years, SD = 1.5) were analyzed. The acceptance rate and finish rate were 89.03 and 99.67%, respectively.

### Reliability analysis

Internal consistency analysis shows that Cronbach’s α of the total scale was 0.942. The dimensions of physiological, psychological and social had a high reliability: 0.915, 0.856 and 0.850, respectively. Cronbach’s α of each factor was between 0.7 and 0.88. The split-half reliability of the total scale was 0.938. The dimensions of physiological, psychological and social were 0.933, 0.890 and 0.881, respectively. The split-half reliability of each factor was between 0.73 and 0.90.

### Validity analysis

The KMO measure of sampling adequacy was 0.947 and the Bartlett test of sphericity was statistically significant (χ^2^ = 7778.7; *P* = 0.000). The method of construct validity was exploratory factor analysis (Varimax). Confirmatory factor analysis (CFA) showed a reasonable fit of the data in the factor structure: χ^2^ = 222,264.171, RMSEA = 0.0536, GFI = 0.867, AGFI = 0.853, CFI = 0.965. There were 12 factors for which the eigenvalue was above 1. The cumulative proportion was 60.53%. Based on scree plot and related theories, 10 factors were extracted. The cumulative proportion was 56.63%. The correlation coefficient between the total scale and sub-scale was high. The dimensions of physiological, psychological and social were 0.929, 0.803, and 0.774, respectively. The correlation coefficient between each factor and its own domain was 0.52–0.89 (Table 2). The correlation coefficient between each item and factor was 0.51–0.88 (Table 3).

**Table 2:** Correlation between factor and dimension

Factors	Physiological	Psychological	Social
F1	0.359	**0.854**	0.475
F2	0.658	**0.788**	0.640
F3	**0.834**	0.460	0.496
F4	**0.794**	0.420	0.421
F5	**0.606**	0.419	0.386
F6	0.578	0.544	**0.891**
F7	**0.795**	0.504	0.510
F8	0.392	0.541	**0.772**
F9	**0.629**	0.354	0.403
**F10**	**0.527**	0.362	0.314

*The bold fonts mean that the factor is highly correlated with the dimension

**Table 3:** Factor matrix and intercommunity

Item	F1	F2	F3	F4	F5	F6	F7	F8	F9	F10	Intercommunity
46	0.612										0.432
47	0.733										0.589
48	0.748										0.635
49	0.564										0.398
50	0.688										0.587
51	0.669										0.518
56	0.596										0.449
57	0.527										0.374
41		0.707									0.720
42		0.738									0.775
43		0.672									0.622
44		0.732									0.687
45		0.658									0.617
52		0.433									0.395
22			0.421								0.462
23			0.450								0.545
24			0.495								0.528
27			0.729								0.711
28			0.765								0.696
29			0.600								0.513
30			0.680								0.586
31				0.671							0.583
32				0.517							0.414
33				0.707							0.624
34				0.693							0.618
35				0.470							0.336
36				0.624							0.487
37				0.590							0.482
3					0.649						0.577
8					0.777						0.661
10					0.794						0.677
11					0.592						0.449
12					0.498						0.350
53						0.702					0.692
54						0.765					0.760
55						0.748					0.746
58						0.666					0.617
14							0.451				0.465
15							0.708				0.607
16							0.611				0.598
17							0.471				0.290
25							0.510				0.580
26							0.464				0.565
4								0.691			0.641
5								0.726			0.672
6								0.757			0.696
7								0.649			0.524
18									0.745		0.621
19									0.639		0.607
20									0.601		0.428
21									0.692		0.521
38										0.709	0.591
39										0.763	0.687
40										0.724	0.603

### Establishing the cut-off point for sub-health among college students

Our previous studies show that the total prevalence of sub-health was between 57.2 and 65.1% [10–13, 17–22]; the prevalence of physiological sub-health was between 42.5 and 55.1%; the prevalence of psychological sub-health was between 32.5 and 35.8%; the prevalence of social sub-health was between 32.5 and 40.7%. The percentage method was used to determine the cut-off point for sub-health in SSS: the reference line of the total scale is 60%, was 50% for the physiological field, was 35% for the psychological field, and was 35% for the social field. The raw score, transformed score and T score of the total scale were 224, 72, and 52, respectively. The raw score, transformed score and T score of the physiological sub-scale were 128, 72 and 50; of the psychological sub-scale, they were 48, 60 and 46; of the social sub-scale, they were 26, 56 and 45, respectively. The lower the score, the worse the health condition (Table 4).

**Table 4:** Cut-off point for the total scale and sub-scale

Percentage	Raw score	Converted score	T score	Clinical evaluation
Cut-off point of physiological sub-health sub-scale				
5	100	50	33	sub-health
15	110	58	39	sub-health
25	116	63	43	sub-health
**50**	**128**	**72**	**50**	**sub-health**
75	139	80	57	health
95	153+	91+	66+	health
Cut-off point of psychological sub-health sub-scale				
5	38	43	33	sub-health
15	43	52	40	sub-health
25	46	57	44	sub-health
**35**	**48**	**60**	**46**	**sub-health**
50	51	66	50	health
75	56	75	57	health
Cut-off point of social sub-health sub-scale				
5	20	44	33	sub-health
15	23	50	39	sub-health
25	25	53	43	sub-health
**35**	**26**	**56**	**45**	**sub-health**
50	28	69	49	health
75	32	75	57	health
Cut-off point of total scale				
5	174	50	34	sub-health
15	190	57	40	sub-health
25	200	61	43	sub-health
50	218	69	50	sub-health
**60**	**224**	**72**	**52**	**sub-health**
75	236	77	57	health
95	260+	87+	66+	health

The boldface in the Table 4 is the cutoff point of the scale or sub-scales

### Fit between the expert evaluation method and scale method

There are five expert evaluated 1000 questionnaires which were randomly selected from the valid 6205 cases. The expert evaluation of sub-health status was performed according to the clinical guidelines for Sub-health published by the China Association of Chinese Medicine [23]. The 5 expert evaluated 200 scales respectively. If the expert cannot sure, the scale will be discussed together. If they are still uncertain, they will interview the student for confirmation. The expert evaluation of 1000 questionnaires showed that the total prevalence of sub-health was 64.49%; physiological sub-health was 47.78%; psychological sub-health was 37.15%; and social sub-health was 36.84%. The fit between expert evaluation method and the scale method was 0.758. The fit in the physiological, psychological and social sub-scale was 0.815, 0.797 and 0.787, respectively.

### The ROC curve to detect the cut-off point of the scale

**Fig. 1:**
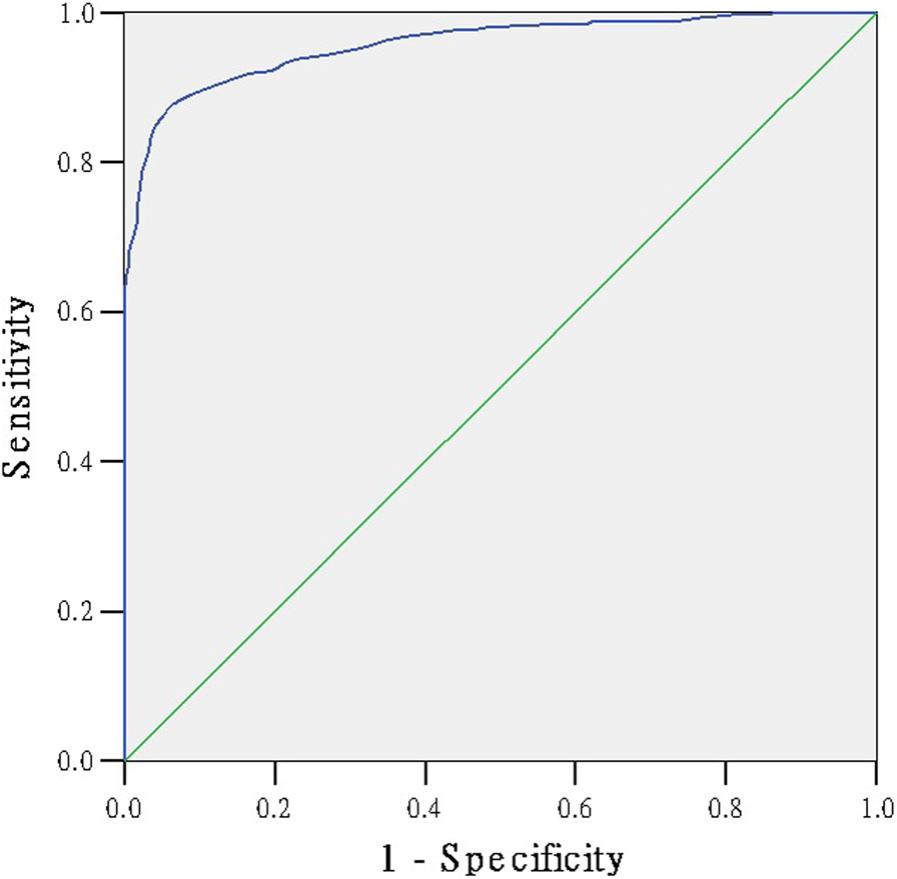
The ROC curve of sub-health status

**Fig. 2:**
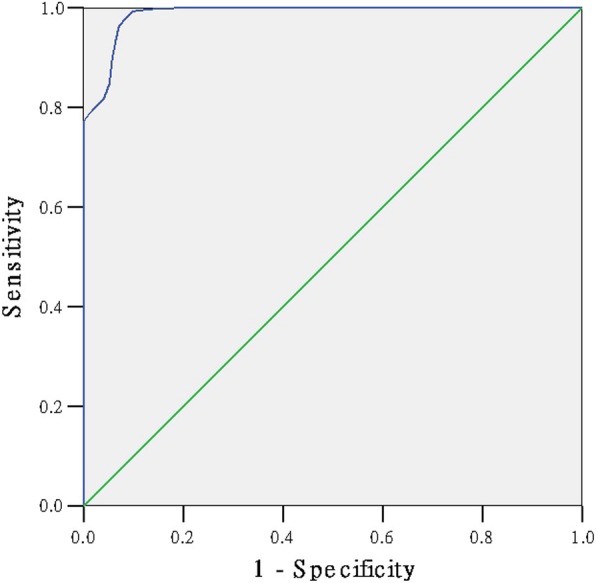
The ROC curve of physiological sub-health status

**Fig. 3:**
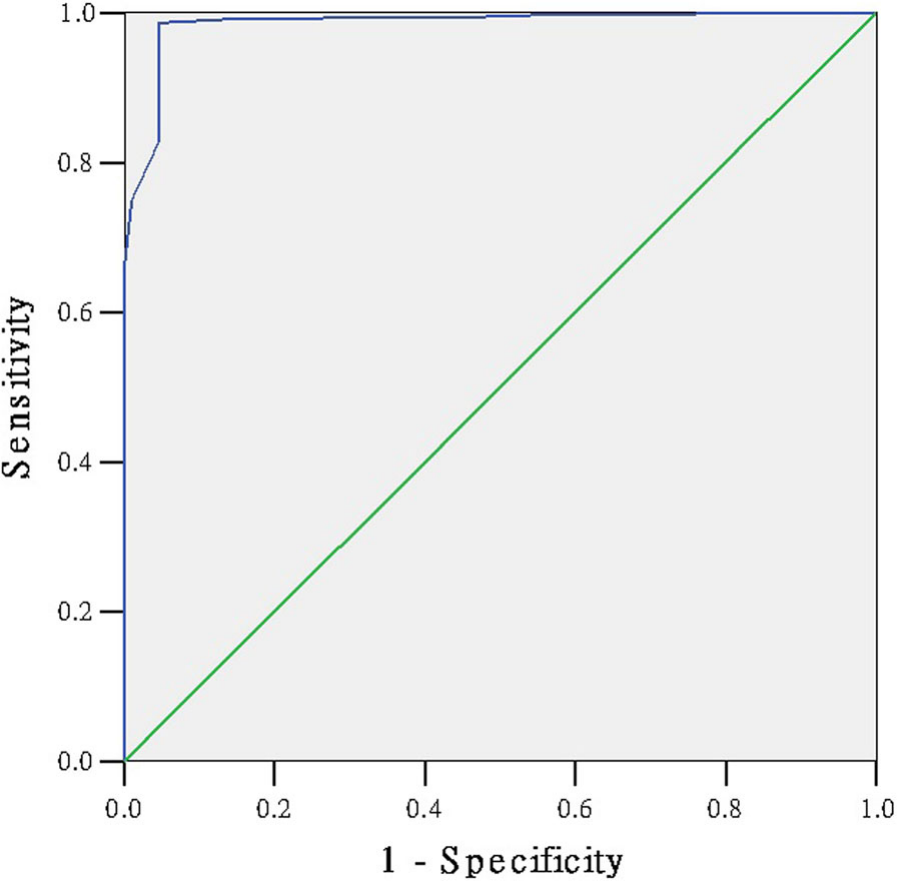
The ROC curve of psychological sub-health status

**Fig. 4:**
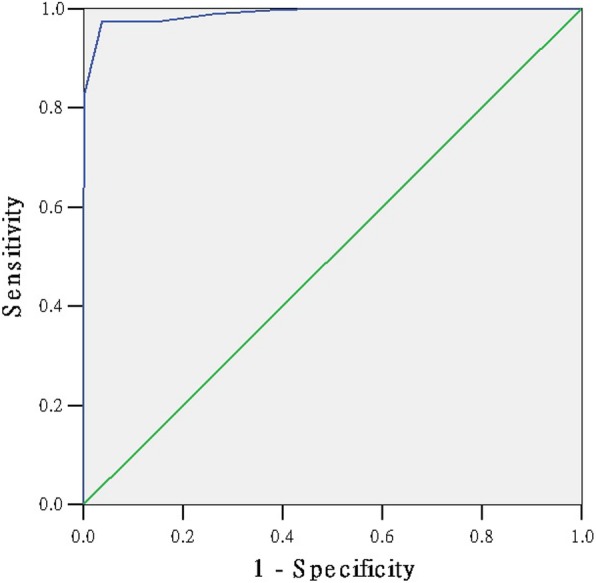
The ROC curve of social sub-health status

We had presented the ROC curve of the 1000 questionnaires to detect the cut-off point of the scale (Figs. 1, 2, 3, 4). The area under the curve in the physiological, psychological, social sub-scale and total scale was 0.987, 0.985, 0.990, 0.958, respectively (Table 5). The SSS was significantly positive to evaluate the sub-health state (*P* = 0.000). Table 6 shows the Youden’s index in each dimension. The maximum value of Youden’s index in the physiological, psychological, social sub-scale and total scale was 0.90, 0.94, 0.94, 0.81 (Table 6). The optimum cut-off point in the physiological, psychological, social sub-scale and total scale was 126.5, 48.5, 25.5 and 221.5. Ultimately, according to the standard above, 2150 were healthy and 3879 were ‘sub-healthy’ in 6029 students.

**Table 5:** the Area Under the Curve

Dimension	Area	Std. Error (a)	Asymptotic Sig. (b)	Asymptotic 95% Confidence Interval	
				Lower Bound	Upper Bound
Physiological	0.987	0.002	0.000	0.983	0.992
Psychological	0.985	0.003	0.000	0.979	0.992
Social	0.990	0.003	0.000	0.984	0.996
total	0.958	0.006	0.000	0.947	0.969

**Table 6:** Coordinates of the Curve

Raw scorce	Sensitivity	1 - Specificity	Youden’s index
physiological sub-health sub-scale			
123.5	0.899554	0.057582	0.841972
124.5	0.928571	0.06334	0.865232
125.5	0.964286	0.071017	0.893268
126.5	0.993304	0.097889	0.895415
127.5	0.997768	0.134357	0.863411
128.5	1	0.180422	0.819578
psychological sub-health sub-scale			
46.5	0.75	0.009852	0.740148
47.5	0.827778	0.045977	0.781801
48.5	0.986111	0.045977	0.940134
49.5	0.991667	0.146141	0.845525
50.5	0.991667	0.228243	0.763424
51.5	0.994444	0.326765	0.667679
social sub-health sub-scale			
23.5	0.575188	0	0.575188
24.5	0.827068	0.001422	0.825645
25.5	0.973684	0.036984	0.9367
26.5	0.973684	0.152205	0.821479
27.5	0.988722	0.260313	0.728409
28.5	0.996241	0.375533	0.620707
total scale			
219.5	0.848967	0.041176	0.80779
220.5	0.864865	0.052941	0.811924
221.5	0.877583	0.064706	0.812878
222.5	0.890302	0.088235	0.802067
223.5	0.898251	0.108824	0.789428
224.5	0.91097	0.144118	0.766852

## Discussion

Sub-health status is considered to be an intermediate status between disease and health. In the traditional Chinese medicine guidelines released by the China Association of Chinese Medicine (CACM), it is characterised by a decline in vitality, in physiological function and in the capacity for adaptation. The prevalence rate of Sub-health status was high (between 57.2 and 65.1% [10–13, 17–22]). Although the prevalence of sub-health is high, there has been a lack of objective clinical diagnostics for it. A number of Sub-health status questionnaires have been established and evaluated in China, such as Suboptimal Health Status Questionnaire (SHSQ)-25, Multidimensional Sub-health Questionnaire of Adolescents (MSQA) and Sub-Health Measurement Scale V1.0 (SHMS V1.0) [14–16]; however, (SHSQ)-25 is targeted at physiological and psychological Sub-health state and MSQA is aimed at adolescents. SHMS V1.0, on the other hand, is also suitable for adolescents. These are not suitable for use with undergraduate students.

The aim of the survey was to develop a valid, simple questionnaire for measuring SHS. Since 2005, our group has been dedicated to developing a sub-health scale. The item pool of the draft questionnaire was discussed in focus groups. WHOQOL-BREF was selected as the validity reference. From a professional perspective and large sample evaluation, the scale ultimately consisted of 58 items. The reliability and validity of the SSS was examined in relation to undergraduate students, and the divided converted score of the total scale was 72.

Firstly, the draft came from a literature review, clinical experience and practice, consultations with experts and closed questionnaires and health-related quality of life scales. The experts in different fields discussed, modified and deleted items, thus ensuring the scale and content of the project more accurately reflected the actual situation of sub-health. The draft scale was revised and modified repeatedly, leading to the final 83 items of the SSS.

Secondly, the draft scale was evaluated in a large sample. Item analysis indicated that the items in the draft SSS had satisfactory sensibility, representation and internal consistency. The index of reliability included Cronbach’s α and split-half reliability, which achieved the psychometric demand. The structure validity achieved excellent levels of content, construct and criterion-related validity. The draft SSS accurately reflected the characteristic of sub-health. As a result, it can be used to evaluate sub-health among undergraduate students. However, the cumulative proportion was not high enough, and the models of SSS need to be revised and reduced. Five methods (dispersion, correlation coefficient, factor analysis, *t*-test and Cronbach’s α) were employed to analyze the 83 items, 63 of which were selected. From a professional perspective, we deleted nine items, which were not representative or have universal application. We added four items for skin factor. The scale ultimately consisted of 58 items.

Thirdly, a large sample survey in China showed that the SSS was highly reliable and valid. In addition, the proportions of acceptance rate (89.03%) and finish rate (99.67%) were high, indicating that participants responded carefully to the questions. The overall Cronbach’s α was good, 0.942. When internal consistency is analyzed in the sub-scales, Cronbach’s α of the three sub-scales was relatively high (0.850–0.915). The homogeneity reliability test showed that reliability coefficients were > 0.70 and total scale reliability coefficients were > 0.90. The scale had high homogeneity. Each factor was highly related to its own domain, and had a low correlation with other domains. The correlation coefficient between the sub-scale and the total scale was also high. The multidimensional structure of SSS was further checked by CFA, and a good fit of the data was observed. The total scale and sub-scales were highly related to the WHOQOL-BREF. The questionnaire was also able to discriminate between healthy and ‘sub-healthy’ undergraduates.

Fourthly, the cut-off point for sub-health in SSS was established by a percentage method. At present, the methods of building the cutoff point of scale, are mainly including dispersion, percentile, index, and related methods. Because each method has different characteristics, generally according to the distribution of the sample and the nature of the scale, different methods are used. The scale is related to the physical and psychological indicators of the human body. The samples of these indicators are normally distributed, also non-normally distributed. The percentile method is suitable for both normally distributed samples and non-normally distributed samples [24, 25]. Therefore, we selected the percentile method to build the cutoff point of scales.

At last, the fit between the expert evaluation method and the scale method was up to 0.70, which is appropriate and valid. The ROC curve of the 1000 selected questionnaires was presented to detect the cut-off point of the scale. Youden’s index is often used in conjunction with ROC analysis. The index is defined for all points of an ROC curve, and the maximum value of the index may be used as a criterion for selecting the optimum cut-off point when a diagnostic test gives a numeric rather than a dichotomous result [26–28]. The maximum value of Youden’s index in psychological and social sub-scale is equal to our percentage method result. Our percentage method result in physiological sub-scale and total scale is close to the maximum value of Youden’s index. The SSS scale can appropriate evaluate the sub-health state of students.

### The limitations

First of all, this scale is only a self-assessment scale, suitable for the student population. Only a few schools have been investigated. The next step is to expand the sample size to investigate more schools and improve the scale. Second, the current There is no objective measurement standard for sub-health. It is difficult to accurately measure the sub-health status only through the self-rating scale. Some people, who is in psychological and social mental illnesses, may be mistaken for sub-health. As a result, we must use a variety of methods and tools to measure sub-health status. For example, combined with current medical history, past history, physical examination results, plus sub-health self-rating scale, anxiety self-rating scale, depression self-rating scale, WHO neurosis screening table, etc. for comprehensive evaluation, and finally obtain more accurate results.

## Conclusion

We established and evaluated a valid instrument, SSS, which encompasses the domains of physiological, psychological and social health, to investigate SHS. The questionnaire is short and easy to complete, and is therefore suitable for use with undergraduate students.

## Additional file

Additional file 1: This is an English language version of the questionnaire for our study. **Table S1.** The reference to questionnaire. (DOCX 18 kb)

## Abbreviations

KMO: Kaiser-Meyer-Olkin.
MSQA: Multidimensional Sub-health Questionnaire of Adolescents
SHMS V1.0: Sub-Health Measurement Scale V1.0
SHS: Sub-health status
SHSQ-25: Suboptimal Health Status Questionnaire-25
SSS: Sub-Health Self-Rating Scale
TCM: Traditional Chinese Medicine
WHOQOL-BREF: the WHO Quality of Life Brief Scale
