# Supplementary material for: The development and evaluation of a sub-health self-rating scale for university students in China
Source: BMC Public Health. 2019 Mar 21;19:330. doi: 10.1186/s12889-019-6650-3 (PMC6429791; doi:10.1186/s12889-019-6650-3)
Supplement: Supplementary file 1 — This is an English language version of the questionnaire for our study. Table S1. The reference to questionnaire. (DOCX 18 kb) [file 12889_2019_6650_MOESM1_ESM.docx]

|  | 1 very good | 2 good | 3 general | 4 little | 5 never |
| --- | --- | --- | --- | --- | --- |
| 1、Are you satisfied with your quality of life in the past 3 months? | 1 | 2 | 3 | 4 | 5 |
| 2、Are you satisfied with your health state in the past 3 months? | 1 | 2 | 3 | 4 | 5 |
| 3、Are you satisfied with your sleep? | 1 | 2 | 3 | 4 | 5 |
| 4、Are you satisfied with your working ability? | 1 | 2 | 3 | 4 | 5 |
| 5、Are you satisfied with your ability to deal with problems? | 1 | 2 | 3 | 4 | 5 |
| 6、Are you satisfied with your social relationships? | 1 | 2 | 3 | 4 | 5 |
| 7、Are you satisfied with the support from your friends? | 1 | 2 | 3 | 4 | 5 |
|  | 1 always | 2 constantly | 3 sometimes | 4 occasionally | 5 never |
| 8、Do you have insomnia? | 1 | 2 | 3 | 4 | 5 |
| 9、Do you find it easy to sleep? | 1 | 2 | 3 | 4 | 5 |
| 10、Do you find it difficult to sleep? | 1 | 2 | 3 | 4 | 5 |
| 11、Do you have many dreams or are easily awakened? | 1 | 2 | 3 | 4 | 5 |
| 12、Do you wake up so early that it affects your rest? | 1 | 2 | 3 | 4 | 5 |
| 13、Do you have enough energy to deal with problems in your life? | 1 | 2 | 3 | 4 | 5 |
| 14、Do you easily feel tired? | 1 | 2 | 3 | 4 | 5 |
| 15、Do you feel muscle soreness, aches and fatigue? | 1 | 2 | 3 | 4 | 5 |
| 16、Do you feel heavy or uneasy in your body? | 1 | 2 | 3 | 4 | 5 |
| 17、Do you get colds easily? | 1 | 2 | 3 | 4 | 5 |
| 18、Do you have rough skin? | 1 | 2 | 3 | 4 | 5 |
| 19、Do you look haggard？ | 1 | 2 | 3 | 4 | 5 |
| 20、Do you have hyperpigmentation or freckles? | 1 | 2 | 3 | 4 | 5 |
| 21、Do you get acne easily? | 1 | 2 | 3 | 4 | 5 |
| 22、Do you suffer from headaches? | 1 | 2 | 3 | 4 | 5 |
| 23、Do you feel your head is heavy? | 1 | 2 | 3 | 4 | 5 |
| 24、Do you feel pain in your shoulders? | 1 | 2 | 3 | 4 | 5 |
| 25、Do you have backache? | 1 | 2 | 3 | 4 | 5 |
| 26、Do you feel leg and knee pain? | 1 | 2 | 3 | 4 | 5 |
| 27、Do you have eyestrain? | 1 | 2 | 3 | 4 | 5 |
| 28、Do you suffer from dry eyes? | 1 | 2 | 3 | 4 | 5 |
| 29、Do you have poor eyesight? | 1 | 2 | 3 | 4 | 5 |
| 30、Do your eyes hurt? | 1 | 2 | 3 | 4 | 5 |
| 31、Do you feel discomfort in your stomach? | 1 | 2 | 3 | 4 | 5 |
| 32、Do you lose your appetite? | 1 | 2 | 3 | 4 | 5 |
| 33、Do you feel bloated? | 1 | 2 | 3 | 4 | 5 |
| 34、Do you get stomach ache? | 1 | 2 | 3 | 4 | 5 |
| 35、Do you have misshapen stools? | 1 | 2 | 3 | 4 | 5 |
| 36、Do you suffer from constipation (less than 3 times a week) or difficult defecation? | 1 | 2 | 3 | 4 | 5 |
| 37、Do you have dry stools? | 1 | 2 | 3 | 4 | 5 |
| 38、Do you have to urinate frequently (more than 3 times a night)? | 1 | 2 | 3 | 4 | 5 |
| 39、Do you feel you never fully empty your bladder? | 1 | 2 | 3 | 4 | 5 |
| 40、Do you feel pain when urinating? | 1 | 2 | 3 | 4 | 5 |
| 41、Do you feel irritable? | 1 | 2 | 3 | 4 | 5 |
| 42、Do you feel oppressed? | 1 | 2 | 3 | 4 | 5 |
| 43、Do you feel lonely? | 1 | 2 | 3 | 4 | 5 |
| 44、Do you feel anxious? | 1 | 2 | 3 | 4 | 5 |
| 45、Do you have negative emotions (e.g. despair, weariness, depression, pessimism)? | 1 | 2 | 3 | 4 | 5 |
| 46、Are you still interested in the things that interested you in the past? | 1 | 2 | 3 | 4 | 5 |
| 47、Do you laugh when you see something funny? | 1 | 2 | 3 | 4 | 5 |
| 48、Do you feel happy? | 1 | 2 | 3 | 4 | 5 |
| 49、Can you relax? | 1 | 2 | 3 | 4 | 5 |
| 50、Are you hopeful about your future? | 1 | 2 | 3 | 4 | 5 |
| 51、Do you feel your life is meaningful? | 1 | 2 | 3 | 4 | 5 |
| 52、Do you easily get nervous? | 1 | 2 | 3 | 4 | 5 |
| 53、Do you find it difficult to concentrate? | 1 | 2 | 3 | 4 | 5 |
| 54、Are you forgetful? | 1 | 2 | 3 | 4 | 5 |
| 55、Do you feel dull? | 1 | 2 | 3 | 4 | 5 |
| 56、Can you undertake your social roles? | 1 | 2 | 3 | 4 | 5 |
| 57、Do you like parties and social events? | 1 | 2 | 3 | 4 | 5 |
| 58、Do you feel your efficiency has decreased? | 1 | 2 | 3 | 4 | 5 |
